# Photosynthetic Machineries in Nano-Systems

**DOI:** 10.2174/1389203715666140327102757

**Published:** 2014-06

**Authors:** László Nagy, Melinda Magyar, Tibor Szabó, Kata Hajdu, Livia Giotta, Márta Dorogi, Francesco Milano

**Affiliations:** 1 Institute of Medical Physics and Informatics, University of Szeged, Rerrich B. tér 1, 6720 Szeged, Hungary;; 2 Department of Material Sciences, University of Salento, Strada per Monteroni, 73100 Lecce, Italy;; 3 Biophotonics R&D Ltd, Szeged, Hungary;; 4 Institute for Physical and Chemical Processes (IPCF), Bari division, Italian National Research Council (CNR), Via Orabona 4, 70126 Bari, Italy

**Keywords:** Bio-nanocomposite, nanosystems, photosynthesis, reaction centre.

## Abstract

Photosynthetic reaction centres are membrane-spanning proteins, found in several classes of autotroph organisms, where a photoinduced charge separation and stabilization takes place with a quantum efficiency close to unity. The protein remains stable and fully functional also when extracted and purified in detergents thereby biotechnological applications are possible, for example, assembling it in nano-structures or in optoelectronic systems. Several types of bionanocomposite materials have been assembled by using reaction centres and different carrier matrices for different purposes in the field of light energy conversion (e.g., photovoltaics) or biosensing (e.g., for specific detection of pesticides). In this review we will summarize the current status of knowledge, the kinds of applications available and the difficulties to be overcome in the different applications. We will also show possible research directions for the close future in this specific field.

## 1. INTRODUCTION

**Fig. (1):**
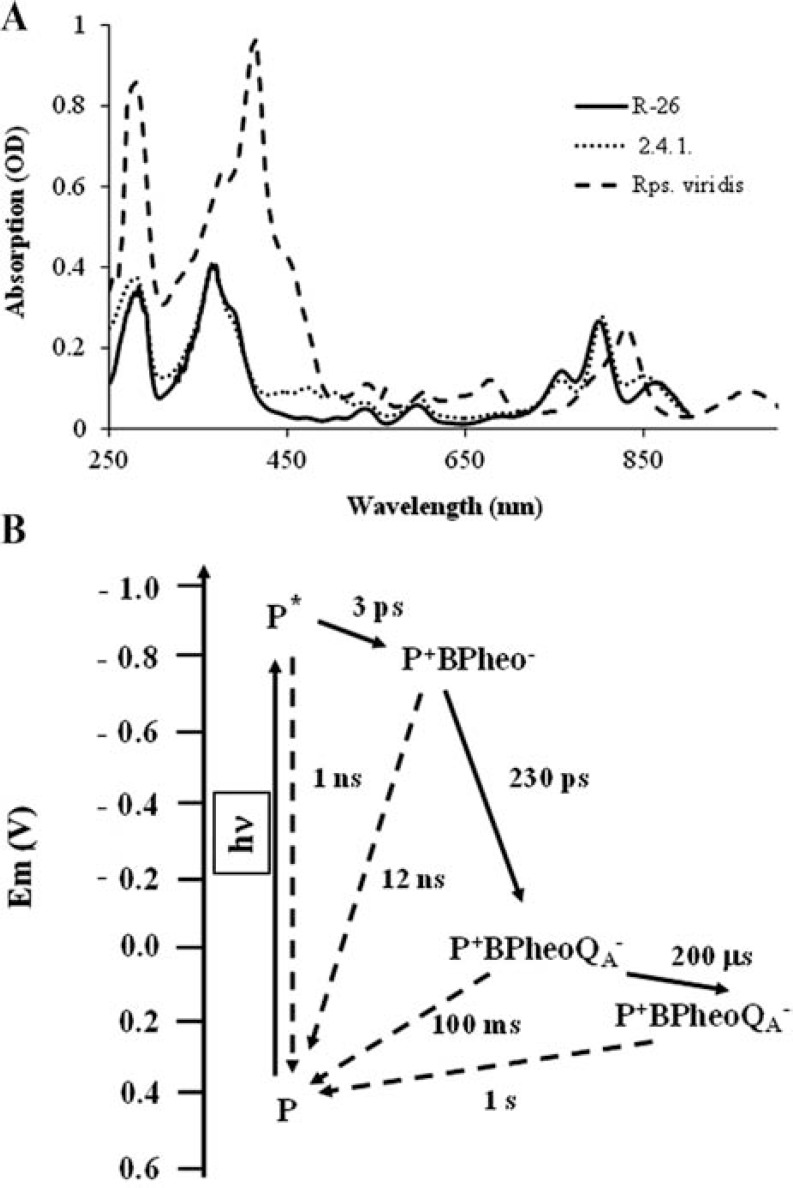
**A)** The steady state absorption spectra of reaction centre proteins purified from three characteristic species of purple bacteria. 2.4.1.: *Rhodobacter sphaeroides* 2.4.1. carotenoid containing strain, usually referred as wild type. It contains BChl-a in the RC protein. R-26: *Rhodobacter sphaeroides* R-26 strain lacking the carotenoids. It, too, contains BChl-a in the RC protein. Rps. viridis: *Rhodopseudomonas viridis*. It contains BChl-b in the RC protein and a bound non-haem type cytochrome subunit. **B)** The reaction pathways of the electron transfer reaction within the reaction centre proteins of *Rhodobacter sphaeroides* species. The redox species and the forward (solid arrows) and recombination (dashed arrows) reactions, the corresponding lifetimes are also indicated.

Photosynthetic reaction centre proteins (RC) are the most efficient light energy converter systems in Nature [1, 2]. The protein possesses such technical properties that unique applications are possible, for example, its use in the nano-structures or in the optoelectronic systems [3]. a) This protein has characteristic light absorption in the near infrared range (700-1000 nm) of the spectrum (Fig. **1A**). It offers applications in equipment using this wavelength range (for example in security devices). b) After the excitation by light there are kinetic components with different lifetimes inside the protein (Fig. **1B**). We can find different kinds of processes from the few femtoseconds of the excitation to the picoseconds of the primary charge separation or to the seconds of the charge recombination. On the whole, such protein can be made that we need, so that it is possible to generate a redox process in any kind of time interval that we are interested in. c) Virtually every absorbed photon is able to generate a charge pair in the RC, i.e., the quantum efficiency is almost 100%. This extreme characteristic of the RCs is very useful for designing systems for harnessing solar energy. d) The redox centres inside the protein after excitation by light can interact with the environment (both on the donor side and the acceptor side). This redox system offers numerous potential applications. In these systems the electron – arising from the charge separation - is trapped in the redox components of the RC or its molecular environment and, among other things, can participate in electric circuits. 

These interesting properties initiated huge efforts for creating bio-nanocomposite materials as well by using RCs and different carrier matrices for several purposes and led to numerous publications in this field. Different types of RCs (bacterial RCs, photosystem I, PSI and photosystem II, PSII of plants) were bound to different carrier matrices (metal electrodes, transitional metal oxides, carbon nanotubes (CNT), etc.) by different methods (physical sorption and specific chemical binding). The aim of these experiments is to create systems for efficient light energy conversion (e.g., photovoltaics), integrated optoelectronic systems or biosensors (e.g., for specific detection of pesticides).

In this paper we would like to overview recent works in this field. We would like to show the current status of our knowledge, the state of the art of applications along with their technical difficulties and limitations. We also try to show possible directions in research for the near future on this specific field.

## 2. PHOTOSYNTHETIC MATERIALS IN BIOTECHNOLOGY

Studying the photosynthetic conversion of light into chemical energy is extremely important in many points of view. Any information we gain about these processes brings us closer to improve sustainable green technology in many fields [4-6]. In food production – in order to increase the photosynthetic production of plants in agriculture it is important to know the intrinsic (controlling the kinetics and energetics of charge separation and stabilization) and extrinsic (photo- and UV inhibition, organic and inorganic pollutants, etc.) limitations of the process. From the point of view of ecology it is important to keep the primary production in ecosystems in the biosphere balanced and there are more and more examples of using photosynthetic materials in bio-remediation – eliminating harmful components (organic agents, heavy metals, etc.) as well. It is interesting to keep in mind that although the RC protein is a real nano-system (its size is 10 nm, and one photon creates one charge pair) it is the energetic basis of virtually all life on Earth (and also the source of the fossil fuels); hence utilization of the solar energy is a real challenge. Based on the unique properties of the photosynthetic systems a new generation of applications (components of integrated optoelectronic devices for photo- and biosensors, fast optical switches and logic gates in circuits, etc.) are also under exploration in bio-nanotechnology research [3]. 

Photosynthetic systems at any level of the organization (from cells and membrane to macro molecular complexes and to simple photosensitive molecules) can be used in smart bio-nanotechnology by combining them with new generations of advanced materials. 

### 2.1. Cells in Biocomposites

Plant, cyano and purple bacterial cells have intrinsic properties that make them very suitable to design hybrid materials (encapsulating them, for example, in organic and inorganic porous matrices) for eco-friendly biosensors and bioreactors [7-10].

Meunier and co-workers encapsulated *Arabidopsis thaliana* plant whole cells into different silica-based matrices. These authors found that even though plant cells were not able to divide within the host matrix, they remain alive for several days without detectable oxidative stress [9]. 

Bacterial cells survive quite well in silica gel. Surprisingly, more bacteria remain culturable in the gel than in an aqueous suspension. The metabolic activity of the bacteria towards glycolysis decreases slowly, but half of the bacteria are still viable after one month. Bacteria can remain alive for many weeks within a porous silica matrix [11, 12]. The enzymatic activity of trapped *E. coli *cells follows the usual Michaelis–Menten law, and their bioactivity was even better than that of the bacteria in suspension [11].

### 2.2. Cell Organelles and Membranes in Bio-Nanotechnology

Chloroplasts and thylakoid membranes extracted from spinach leaves suspended in suitable aqueous buffers can be used as sensing elements for optical detection of specific photosynthetic herbicides like atrazine, terbutryn and diuron, as well as of copper and mercury bivalent cations [13-15].

Ventrella and co-workers functionalized quartz (Qz) substrates by layer-by-layer (LbL) method using PSII-enriched thylakoid membrane fractions (BBY) obtained from spinach leaves and poly(ethylenimine) (PEI) as polyelectrolyte to modify the substrate [16]. The activity of the hybrid material was checked by adapting the Hill Reaction [17] optical assay to solid substrates. The authors reported that the activity of the biomaterial was preserved at 85–90% after immobilization. The BBY modified Qz supports were used to test their ability to sense the triazine type herbicide, terbutryn. The chips allowed terbutryn to be detected at concentrations higher than 1.58×10^−7^ M [16]. This value is close to the regulation limit that is currently fixed by EU for pesticide concentration in drinking water at 0.1 ìM [18].

Patches of photosynthetic membrane from *Rhodobacter (Rb.) sphaeroides* were connected to a bare-gold surface and the adhered membranes remained functional for up to 3 days during continuous illumination under ambient conditions [19].

### 2.3. Photosynthetic Reaction Centres in Bio-Nanotechnology

Authors certainly admit that the use of whole cells and membranes, as active parts of bio-composites, instead of proteins, could prove very useful in various applications. The purification of membrane proteins and enzymes are usually complicated, time consuming processes, which costs more than the production of cells. Nevertheless, isolated proteins have advantages in many cases. They bind specific cofactors to specific sites effectively and react with them. Also, there are no compensating metabolic processes. For this reason, it is definitely advantageous to study possible roles of specific proteins in bio-nanotechnology. The aim of this paper is to discuss the conditions and limitations of selected possible applications of photosynthetic reaction proteins in nano-systems. 

## 3. RCS IN BIO-NANOCOPOSITES

Based on the unique properties of the RCs [3], possibility for different applications can be visualized and have also already been explored so far. Preliminary demonstrations are as possible photosensors [20-23], optoelectronic components [24, 25], gating elements for phototransistors [21, 22] and biosensors for the detection of herbicides [15, 18, 26] and other environmental pollutants [27]. A distinguished interest is addressed to possible applications as photovoltaic devices [28, 29] and light-powered fuel cells [30].

### 3.1. Different Types of Reaction Centre Proteins are Used

RC is a pigment protein complex, which converts the energy of light into chemical potential of charge pairs with extremely high efficiency. The quantum yield of the primary charge separation is almost 100% [31], which could not be reproduced by humans up to now. This very efficient charge separation is warranted by specific structure and function. Although, there are several types of RCs in living beings (PSI, PSII of plants, algae and cyanobacteria, RCs of purple and green bacteria; [32]), possibly developed from the ancient monomer protein, the basic processes are essentially the same. These include (a) electron excitation by light, (b) charge separation and stabilization, (c) rearrangement of the dielectric medium and hydrogen bond interactions (including protonation and deprotonation of specific amino acids), (d) conformational movements within the protein (including transition of (sub)states between dark and light adapted forms, and relaxation processes) and (e) receiving and sending electrons from and to the redox carriers in the environment under proper conditions.

Based on these common features different types of RC proteins purified from various organisms are used in many laboratories for applications in bio-nanotechnology. Photocurrents generation have been reported, for example, for PSI and PSII RCs from plants and/or cyanobacteria [21, 30, 33-40] and from purple bacterial RCs isolated mainly from *Rb. sphaeroides* [29, 33, 41-51]. RC-LH1 complexes of purple bacteria comprising the native RC and light-harvesting (LH1) proteins are also used for this purpose [52, 53].

### 3.2. Methods in RC-Biotechnology Research

#### 3.2.1. Immobilization Techniques

**Fig. (2):**
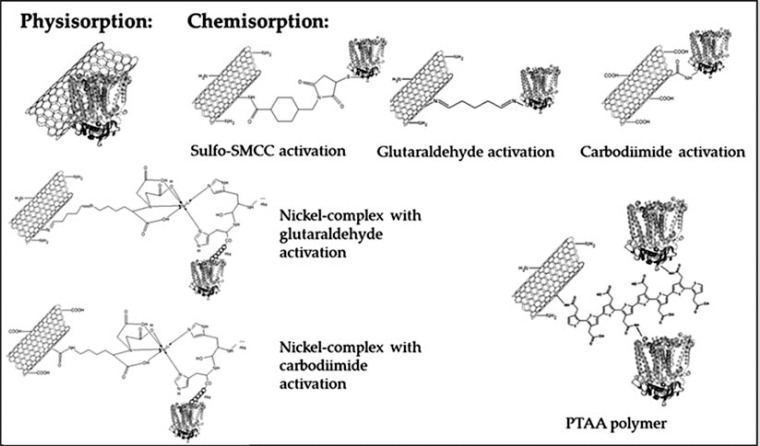
Summary of the binding procedures of RCs to carbon nanotubes. The figure shows physical non-covalent hydrophobic binding to nonfunctionalized CNTs and to functionalized nanotubes through different functionalization procedures and with crosslinkers.

The exceptional characteristics of bio-nanocomposites require special interaction between the bio- and carrier matrices. Several types of interactions can be investigated, among others, photosensitization (direct energy transfer) between the chromophores, electrostatic interactions between the components or transfer of electrons to or from conducting materials. In order to assure these basic functions of the nanodevices several immobilization techniques are developed (Fig. **2**).

##### Physical Sorption

The simplest, nevertheless, in many cases, the most efficient technique is a physisorption onto different substrates [54-57]. There is ambiguous information in the literature about the efficiency of the complexes prepared by this method. The most important concerns about this method are that on the bare inorganic surface the protein structure as well as the orientation is not known. Considerable conformation change is also expected which modifies the accessibility of the active centres, the specific binding surfaces and/or the redox properties of the intraprotein cofactors. Although these concerns should be appreciated, there are many proofs that different redox proteins remained functional on bare-gold (yeast cytochrome [58]), CNTs [54] or indium tin oxide (ITO) [57] electrodes surfaces. Surprisingly high photocurrent was measured by Hollander and co-workers [59] when RCs of *Rb. sphaeroides* and RC-LH1 complexes from *Rhodopseudomonas (Rps.) acidophila* were adhered to bare-gold electrodes. It should be kept in mind that yeast cytochrome has one surface exposed cysteine which is a good candidate to attach to the bare-gold surface through covalent binding [58].

**Fig. (3):**
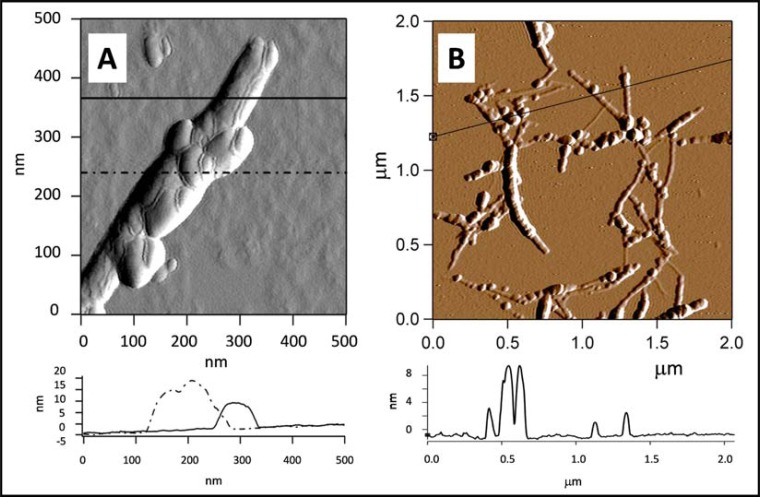
AFM amplitude image and height section of photosynthetic RCs sitting on MWNT after physical binding [62] (A) and after chemical crosslinking to amine functionalized SWNT by GTA (B, kindly provided by Ms. K. Nagy and Dr. Gy. Váró, MTA BRC, Szeged, Hungary).

For attaching proteins to bare-electrode surface the hydrophobicity of the electrode should also be taken into account. While fluorine doped tin oxide (FTO) and ITO surfaces are hydrophilic, Pt and CNTs are hydrophobic. Since RCs are transmembrane proteins a strong hydrophobic interaction could account for the driving force for efficient binding to inert surfaces [54, 60]. The AFM amplitude and height section image demonstrate examples that it is possible to attach RCs to multi-walled carbon nanotube, MWCNT, after physical binding (Fig. **3A**) and crosslinking chemically to single walled carbon nanotube, SWCNT (Fig. **3B**, kindly provided by Ms. K. Nagy and Dr. Gy. Váró, MTA BRC, Szeged, Hungary). 

Although the structural details (orientation, conformational changes) are unknown in these samples the photochemical/-physical activity of the complexes proves efficient functional binding. Absorption change at specific wavelengths after saturating flash excitation indicates stabilization of light induced charges in RCs attached to CNTs by physical binding [54]. Conductivity in a two electrode electrochemical cell was changed after series of flashes when RCs were deposited on Pt working electrode [60]. 

##### Chemical Binding

In order to investigate specific interactions specialized chemical (covalent) binding can be achieved by several strategies. Common or engineered functional groups (like amine, carboxyl or sulfhydryl) or specialized cross linkers can be used [61]. 

One trivial way of the binding is using amine and carboxyl functional groups on the protein, however, one should take into consideration that specific activation is necessary which might lead to decrease in functional activity. On the other hand, the binding orientation will not be specific.

The RCs keep their activity if bound to amine or carboxyl functionalized CNTs [62, 63], however, other binding strategies can be useful to increase the activity of the complex. 

Several homo- (e.g., glutaraldehyde) and hetero-bifunctional (e.g., sulfo-SMCC, *N*-(1-Pyrene)iodoacetamide) crosslinkers can be used to bind specific and nonspecific sites of the RCs to different carrier matrices, e.g., to CNTs [63], highly ordered pyrolytic graphite (HOPG [64]) or porous silicon [65]. 

A promising approach is to bind the RCs to transparent conducting electrode (e.g., ITO) in a photo-electrochemical cell through conducting polymer (Poly(3,4-ethylenedioxy-thiophene) (PEDOT), Poly bis(4-phenyl)(2,4,6-trimethyl-phenyl)amine (PTAA)) [62, 66]. The conducting polymer connects larger amount of RCs and wires electrons to the working electrode. The RC/PTAA/CNT complex showed photochemical activity as indicated by flash photolysis experiments and a photocurrent was measured which was sensitive to the mediator applied [66].

##### Self-assembly of Monolayer of Small Molecules

It is very common in the literature that the functions of proteins bound to bare-metal surface or through chemical binding are perturbed [33, 43, 67, 68]. The protein structures are changed, often degraded, and the redox properties of the cofactors are altered. The orientation is usually not controlled and the specific active surfaces can be hindered – unless activity measurements show it otherwise. To avoid these disadvantageous structural and functional changes an appropriate self-assembling monolayer (SAM) of organic molecules are used to coat the metal working electrode for providing a specific surface to which the protein will adhere (ref [29] for a review). 

For example, a very small photocurrent from *Rb. sphaeroides* RCs was measured when RCs were adhered to bare-Pt electrode in the presence of cytochrome-c, but it was enhanced substantially when the electrode was precoated with SAM [69]. The complex of RC-LH1 proteins also showed different adherence when it was bound to SAM coated gold electrode compared to bare-gold surface [52]. 

In addition to the stimulating effect of the SAM through assuring the proper conformation stability it serves as an insulator that reduces the rate of electron tunnelling between the electrode surface and the redox centres of the RC [49]. The effect of the distance from the electrode surface to the redox centres of the protein on the electron tunnelling is well demonstrated by using PSI, for example [48] or by Hollander and co-workers [59].

These later authors used mercaptopropanoic (C2) and mercaptoundecanoic (C10) acids for covering bare-gold electrodes to create SAM and measured photocurrent generation after binding the RCs to the modified surface. Authors found lower photocurrent generation when C10-SAM was used. There are several trivial interpretations of the low current: lower binding, unfavourable orientation, different structures on different surfaces. This finding, however, fits the earlier results of Trammel and co-workers [48] who found a considerable drop in photocurrent when the distance from the RC to the electrode surface exceeded 1 nm. Although the trivial losses cannot be excluded, the thickness of C10-SAM is larger than this threshold. As a result, the enhancing compensating and decelerating effects should be balanced when experiments are planned.

##### Functionalization by Polyhistidine Tag

An appropriate SAM functionalization is to bind RC proteins labelled by engineered polyhistidine tag. Since, this labelling allows binding to specific sites the effect of the orientation can be examined [33-35, 43, 48, 49, 70-73]. The specific reaction of the poly-His tag is to create chelate complex with terminal nitrilotriacetic acid (NTA) supported by Ni^2+^. Consequently the prerequisite of this type of binding requires decorating the inorganic surface with this functional group [43]. CNTs functionalized either by COOH or –NH_2_ groups can also be labelled by NTA-Ni^2+^-poly-His decoration, consequently RCs can be bound (Fig. **2**, [74]). 

##### Specific Cysteines

The unique role of cysteine residues at specific sites of the RCs is discussed in detail by Hollander and co-workers [59]. On the one hand creating disulphide bridges between proteins and bare-gold surfaces is a common and not so difficult way for immobilization. On the other hand specific oriented binding of the protein can be achieved by the known location of cysteines in the RC structure. 

There are several indications that surface-exposed cysteine residues of proteins play essential role for the binding. One example can be the native PSI complexes of *Synechocystis* sp., which does not contain this type of cysteine and does not bind to gold electrode. However when this type of cysteine is introduced by genetic engineering the adherence is shown [75].

Another bright example can be the difference in binding of RC-LH1 complexes isolated from *Rps. palustris* [53] and *Rps. acidophila* (see discussion in [59]). It is possible that the RC tetraheme cytochrome in the *Rps. acidophila* RC-LH1 complexes is bound to gold electrode through cysteine at specific site and wires the electron from the electrode to the RC cofactors. 

#### 3.2.2. Measurement of Photosynthetic Activity

##### Optical Assays

RCs isolated from *Rb. sphaeroides* show several absorption peaks in the range 350-900 nm rising from the cofactors (pigments) embedded in the protein scaffolding (ref. to Fig. **1**). Upon photo-induced generation of the charge separated state, the protein optical spectrum changes in a considerable way, mainly due to the disappearance of the absorption peak assigned to the dimer (primary donor) at 865 nm, but changes are observed in the whole spectrum. Monitoring the photosynthetic activity is hence relatively simple both in kinetic and steady state mode. In kinetic mode, a short and intense saturating light pulse is used for generating the charge separated state and then the relaxation to the neutral state is recorded. The kinetic features of this relaxation provide information on the activity of the secondary quinone acceptor, Q_B_ site, which is the target of several classes of herbicides and can be used for sensing purposes. Fig. (**4**) shows as examples the binding of atrazine and terbutryne to the Q_B_-site of the reaction centre of *Blastochloris viridis*. This figure was drawn by using the appropriate crystal structures in the presence of these herbicides downloaded from the Brookhaven Protein Data Bank. It demonstrates that besides subtle differences in the biding structures, these chemicals bind essentially to the same area in the protein being in competition with the secondary quinone. Depending on the bacterial species and the kinetic model, which includes the contributions and lifetimes of the charge recombination and those of the quinone binding/unbinding to the protein, the occupation of the site can be calculated [76]. In steady state mode a constant flux of light illuminates the RCs generating a constant amount of charge-separated state: in this case a variation of the Q_B_ site activity is seen as increase (more Q_B_ activity) or decrease (less Q_B_ activity) of the signal associated to the charge separated state.

When dealing with whole cells, the so-called fluorescence induction is more convenient to measure. Upon illumination with a CW 800 nm laser diode, a fluorescence originating from bacteriochlorophylls can be detected in the NIR at λ>850 nm. In the kinetics mode, two phases are clearly distinguishable: a sharp rise, representing the constant or dark fluorescence (F_0_), arising from bacteriochlorophylls unconnected to the RC, and a slowly increasing phase representing the time-dependent variable fluorescence, F_v_(t), raising to a maximum level F_max_ = F_0_ + F_v_ originating from antenna molecules connected to the RCs. Therefore the F_v_/F_0_ ratio measures the efficiency of the photochemical trapping of the absorbed photons and can be related to the photo-conversion efficiency of the RCs [77]. Similar assays are used also for plant photosystems for which a direct observation of the charge separated states cannot be readily achieved.

An optical assay of photosynthetic activity of plant photosystems have also been developed using 2,6-dichlorophenolindophenol, (DCPIP, or DPIP) which has a higher affinity for electrons than ferredoxin and the photosynthetic electron transport chain can reduce DCPIP as a substitute for NADP^+^, that is normally the terminal electron acceptor. Since DCPIP is blue with a maximal absorption at 600 nm in its oxidized form and when reduced becomes colourless, the photosystem activity can be easily followed as the disappearance of the 600 nm band. The assay was applied by Hill for suspension [17] and further developed for solid bio-functionalized substrates [16].

##### Electric Measurements 

For electric measurements transparent conducting oxides (TCO) thin films, like ITO are excellent candidates. These materials are typically used in devices where these properties are essential, like LCD, OLED and electrochromic displays, in touch panel and antistatic window coatings, etc. 

Due to their special band structure [78] TCO and ITO as well, have special optical and electric properties. The conductivity and transmission of the ITO depends on the environmental conditions (humidity, oxygen tension, pollutants, etc.), on the way it was produced, on layer thickness, etc. It has large transparency in the visible range and can be excited by blue and UV light due to a direct and indirect band gap of about 3.7 and 2.8 eV, respectively [78]. 

A unique application is offered by combining them with photosynthetic materials via providing electric contacts or electrodes in electrochemical cells [29, 44, 57]. 

Szabó and co-workers reported a change in the electric resistance when RCs of *Rb. sphaeroides* were deposited and dried onto ITO and illuminated with blue light (about 400 nm) [57]. No photo-conductance was observed by red light excitation which excites only the RC protein. It can thus be concluded that the excitation of both the RC and the ITO is necessary to measure photo-conductance. The ITO/RC system is a good model for optoelectronic application. This system shows light induced change in the resistance even in the dried form.

##### Photoelectrochemical Cells

One of the main aims of the research with RCs in many laboratories is generating photocurrent in an electrochemical cell. There are many types of photo-electrochemical cells depending on the research strategies. One strategy is to dissolve RCs on the bulk electrolyte and shuttle the donor and acceptor side to the cathode and anode by redox mediators [79]. The direction of these studies is to find the best electrode and mediator combinations and a larger absorption cross section to obtain larger photocurrent.

The other strategy is to bind the RC to the surface of the working electrode by one of the methods mentioned above (in paragraph “3.2.1. Immobilization techniques”). The steady current should depend on the absorption cross section, charge stabilization (the rate of charge separation and recombination), electron tunnelling from the protein to the electrode (which depends on the orientation and the distance between the electrode and the redox centres), and the charge carrier ability of the mediator (binding and unbinding turnover rate, diffusibility). 

Different materials can be used for the electrode, for example HOPG [64], and bare- or SAM-modified gold [29, 59]. A unique possibility is to enhance the chromophore concentration by binding the protein to porous materials prepared from nanoporous TiO_2_ [80] or mesoporous WO_3_–TiO_2_ [44] films. The 3D arrangement of the porous materials provides larger surface area, consequently larger chromophore concentration in a unit apparent surface of the electrode. Lebedev and co-workers designed a special arrangement by binding RCs into the inner surface of the wall of the CNTs [67]. This arrangement provides not only increased coverage of the specific surface area of CNTs but excellent possibility for orientation of this hybrid material for efficient photocurrent generation. In addition to the increased absorption cross section, due to the larger chromophore concentration, the photonic structure of the whole material is modified as well. This alteration of the photonic structure with the RC binding is well demonstrated when RC is bound to porous silicon (PSI [65]). The change in the photonic structure after the protein binding is well demonstrated by shifting the specific mode in the reflection spectrum of the RC/PSi complex.

**Fig. (5):**
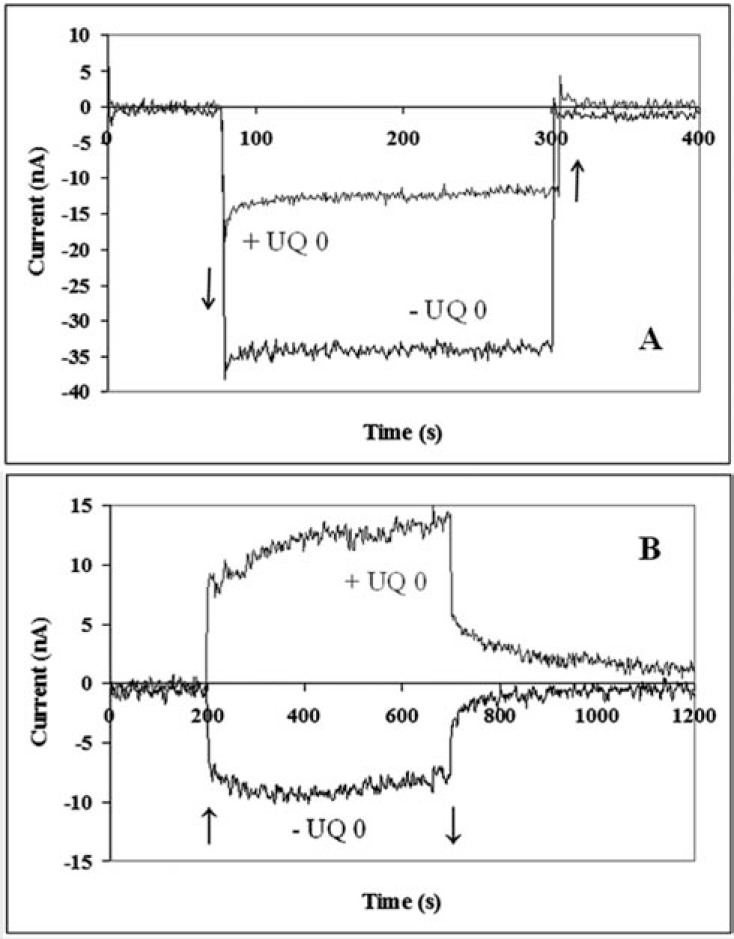
Light induced photocurrent of the electrochemical cell using PTAA covered ITO electrode without (A) and with bound RC (B). UQ 0 mediator was added and light was switched on (↑) and off (↓) as indicated by the arrows [61].

Two [80] and three-electrode configurations [66, 61] are used. In the three-electrode configuration the photocurrent can be measured either at the open circuit or at the applied electrode potential [50]. The measured current was sensitive to the mediator, which indicates the involvement of the RCs in the redox processes (Fig. **5** [66]). 

### 3.3. Possible Directions of Practical Applications

#### 3.3.1. Integrated Optical Devices

If integrated with solid state electronics, photosynthetic complexes might offer an attractive architecture for future generations of circuitry where molecular components are organized by a macromolecular scaffold. But, like other protein molecular complexes, photosynthetic complexes are soft materials, optimized for operation in a lipid bilayer interface between aqueous phases. For utilization in practical technological devices they must be stabilized and integrated with solid-state electronics. Such stabilization was obtained for example by Das and co-workers [33] by using surfactant peptides, and then coating the photosynthetic proteins (both spinach PSI and bacterial RC) with a protective organic semiconductor.

Future realization of protein-based bio-nanocomposite materials may serve as conceptual revolution in the development of integrated optical devices, e.g., optical switches, micro-imaging systems, sensors, telecommunications technologies or light energy harvesting [81-83].

The special properties of the RC [3] allow several possible applications in integrated optical devices. This possibility relies especially on the fact that the electron-hole pair is formed after photoexcitation very quickly, within about 1 ps. It is thought that photosynthetic systems resemble the characteristics of photodiodes and type-II semiconductors, because of the extremely rapid charge separation in space due to the built-in potential. However, the definite advantages of these hybrid systems prepared by using photosynthetic materials are three-dimensional self-assembled systems as compared to the solid state devices [20].

An ultrafast, subpicosecond photonic switch was demonstrated to operate by using the transition of specific sub-states of the chromophore of the bacteriorhodopsin coupled to an optical device (Mach-Zehnder interferometer, [81]). RC protein performs also ultrafast optical transitions (change in absorption and refractive index [84, 85]), so, theoretically it is suitable for similar device.

The newest approach to overcome silicon-based electronic devices is the exploit of molecular electronics in which single (macro-)molecules are used as active elements in nanodevices [86]. Among the various possibilities (AFM conducting tips, nanopores), nanogap electrodes, in particular, seem to be promising because they do not require feedback to maintain the arrangement (comparing with conducting tip AFM) and are less stochastic with respect to nanopores structures. Nanogaps electrodes (namely, a pair of electrodes with a nanometer gap) can be fabricated by electromigration with precise control of the gap properties and are activated by the insertion of specific molecules between the electrodes [87]. In a recent work the photoactive proteins bacteriorhodopsin (Br) and *Rb. sphaeroides* RCs have been used as active materials in nanogaps [88]. Shown in Fig. (**6**), a nanosized optoelectronic device core activated by a single protein molecule is depicted.

It is important to stress that in such kind of device one or few molecules are actually present between the electrodes. In the case of RCs, a resonant tunnelling response can be recorded at 2.5 V in the dark, while under light irradiation no current is detected because the charge separation inside the protein does not allow current transmission. In the case of Br a photo-induced voltage of 50-60 mV was detected upon illumination of the system with green light, which excites the main Br absorption band, while there was no response with blue or red light. Possible application of this kind of device is in sensing, imaging, and solar energy conversion.

#### 3.3.2. Photovoltaics

The energetic demands of the modern society, and the awareness that fossil fuels are exhaustible, bring about the necessity of producing power from renewable sources. The most obvious source of power is sunlight, considering that the solar energy incident on the surface of Earth in one hour is more than that used by the mankind in one year. At present, commercially available solar photovoltaic cells are based on mono and multi-crystalline silicon and have an efficiency of around 20%. However their production requires high temperatures (up to 1400°C) and high vacuum conditions, leading to high manufacturing costs. Bulk-heterojunction organic solar cells are seen as the next commercial solar cells generation with much less production costs, but at the moment they do not meet the efficiency and durability requirements for the general market. Moreover they absorb only in the visible region of the spectrum (300-700 nm) limiting their capability to convert the incoming solar radiation. Another limitation for the organic-based solar cells is the small diffusion range of the photo-generated excitons (typically 10 nm) compared to the layer thickness needed for harvesting a suitable amount of light (typically > 100 nm). 

Photosynthetic complexes such as PSI and bacterial RCs offer possible solutions to these problems. Better harvesting of the sunlight could be obtained using low-band-gap protein complexes such as bacterial RCs, which allow capturing energy in the 300-1000 nm range. The yield of conversion of visible light in electric power can be further increased by covalent binding of organic antennas to the protein scaffolding of the photosystems in close proximity of the pigments in order to allow Förster resonance energy transfer (FRET) to occur, as recently demonstrated by Milano and co-workers [89]. Moreover, thanks to the intrinsic quantum yield of more than 95%, the conversion efficiency of a solar cell based on these proteins can reach or exceed 20%.

The key point of using photosynthetic material for generating power is the production of hole-electron pairs that have to reach the electrodes and to generate photocurrent. For this reason, it is important to make an intimate contact between the RCs and the electrode and to have the proteins oriented in the correct way. It is widely accepted that SAM coating of the electrode helps the protein adhesion on the electrode and prevents the protein denaturation, but has the drawback of somewhat insulating the protein from the electrode. As a consequence, the majority of studies of photocurrent generation have reported current densities on the order of a few hundred nA/cm^2^ [46, 48, 50]. A significant improvement has been obtained by incorporating RCs into mesoporous metal oxide films, resulting current densities up to 120 μA/cm^2^ [45]. Remarkably, photocurrents of the order of μA/cm^2^ were obtained by adhering native *Rps. acidophila* RC-LH1 complex on bare-gold electrodes directly [59], demonstrating for the first time that functionalization of metal electrodes is not necessarily required to obtain high photocurrents and greatly simplified fabrication procedures can be used.

#### 3.3.3. Sensors

Biosensing is one of the many possible practical applications of biomolecules. In the case of photosynthetic proteins, the most obvious targets of biosensing are herbicides [18] although different targets have also been tested, e.g., heavy metals [14, 15]. The classical approach for the herbicide detection makes use of antibodies, which suffer from many limitations, especially the antibodies against small molecules, since their generation is a time consuming process, requires animal models and also blocking in immunoassays is a complex problem [90]. On the other hand, photosynthetic biosensors can be used to detect a wide range of herbicides, either as whole organism, or isolated RCs and membranes. 

In the typical case of an electrochemical transducer, controlled immobilization of the biomolecules on the electrode is the key point. One of the most promising approaches is to modify the electrode surface by means of organic SAMs since the thickness of the organic layers and surface properties are adjustable to suit different sensing applications.

The various class of herbicides, such as triazines (e.g., atrazine), triazinones (e.g., metribuzin), phenylureas (e.g., diuron) and phenols (e.g., bromoxynil) have a common mode of action based on the inhibition of PS-II or PS-II-like purple bacterial RCs. They interact with the D1 protein of PS-II or with the L subunit of the purple bacterial RC, replacing the secondary electron acceptor plastoquinone or ubiquinone from its binding site (Q_B_) [91]. The sensitivity towards heavy metals, e.g., Hg, Cr, Cd, Pb, Zn, Ni and Cu, depends on the nature of the metal and has not been unambiguously determined. Nevertheless, for plant photosynthetic material heavy metals always diminish the photosynthetic activity, which can be detected in several ways: oxygen evolution by Clark electrode, optical detection of reduced form of a suitable acceptor, such as DCPIP, amperometric detection in an electrochemical cell, etc. [13]. For bacterial RCs, the analyte can be detected as reduced photocurrent in an electrochemical cell, or, for the case of herbicides, as faster recovery of the photooxidized dimer at 865 nm [92] or a reduced amplitude of the photobleaching of the same band. 

## Figures and Tables

**Fig. (4):**
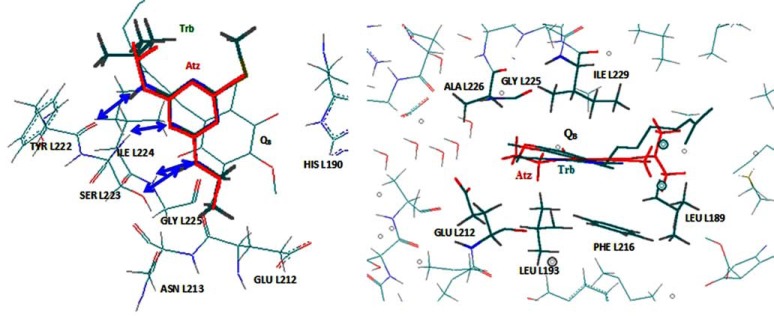
The binding of atrazine and terbutryne (as indicated) to the reaction centre of *Blastochloris *(formerly *Rhodopseudomonas*) *viridis *according to the crystal structure. The figure was drawn by using the files downloaded from the Brookhaven Protein Data Bank. The pdb codes are: 2prc (RC/ubiquinone), 1dxr (RC/terbutryne), 5prc (RC/atrazine) complexes. The arrows show the possible hydrogen bonds (the distances shorter than 3 Å) and neighboring amino acid groups. The graph was realized by HyperChem 7.0.

**Fig. (6):**
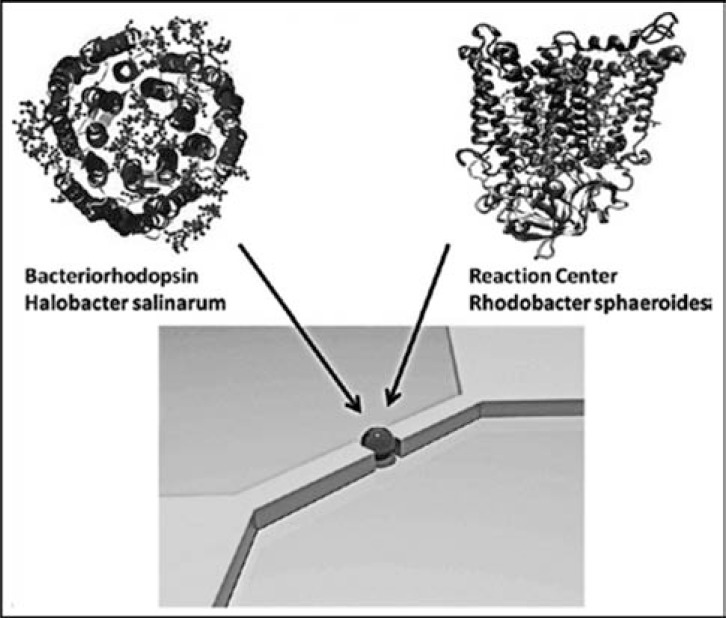
Schematic representation of a nanosized optoelectronic device: the two electrodes are spaced by a few nanometers and the contact can be made by the insertion of a single protein molecule of either bacteriorhodopsin or RC. After small modification from [88].
